# Factors influencing antibiotic prescribing for respiratory tract infections in primary care – a comparison of physicians with different antibiotic prescribing rates

**DOI:** 10.1080/02813432.2024.2332757

**Published:** 2024-04-16

**Authors:** Olof Cronberg, Mia Tyrstrup, Kim Ekblom, Katarina Hedin

**Affiliations:** a Växjöhälsan Primary Healthcare Center, Växjö, Sweden; b Department of Research and Development, Region Kronoberg, Växjö, Sweden; c Department of Clinical Sciences in Malmö, Family Medicine, Lund University, Malmö, Sweden; d Lundbergsgatan Primary Health Care Centre, Malmö, Sweden; e Department of Medical Biosciences, Clinical Chemistry, Umeå University, Umeå, Sweden; f Futurum, Region Jönköping County, Sweden; g Department of Medical and Health Sciences, Linköping University, Linköping, Sweden

**Keywords:** Antibiotic prescribing, diagnosis-linked prescription, infectious disease, physicians’ behaviour, point-of-care testing

## Abstract

**Background:**

There has been a notable decrease in antibiotic prescribing in the last thirty years in Sweden. Little is known about factors influencing antibiotic prescribing over several years.

**Objective:**

To compare primary care physicians who, over time, reduced their antibiotic prescribing for respiratory tract infections with those who remained either high or low prescribers regarding potentially influencing factors.

**Design and setting:**

A register-based study including all RTI visits in primary care in Region Kronoberg, Sweden 2006–2014. The data were divided into three 3-year periods.

**Subjects:**

The data comprised all physicians who had diagnosed at least one RTI for each of the three-year periods. The antibiotic prescribing rate adjusted for the patients’ sex and age group was calculated for each physician and period, and based on the change between the first and the third period, the physicians were divided into three prescriber groups: The High Prescribing Group, the Decreasing Prescribing Group, and the Low Prescribing Group.

**Main outcome measures:**

For the three prescriber groups, we compared factors influencing antibiotic prescribing such as the characteristics of the physicians, their use of point-of-care tests, their choice of diagnoses, and whether the patients returned and received antibiotics.

**Results:**

The High Prescribing Group ordered more point-of-care tests, registered more potential bacterial diagnoses, prescribed antibiotics at lower C-reactive protein levels, and prescribed antibiotics more often despite negative group A Streptococci test than in the Low Prescribing Group. The Decreasing Prescribing Group was between the High Prescribing Group and the Low Prescribing Group regarding these variables. The lower prescription rate in the Low Prescribing Group did not result in more return visits or new antibiotic prescriptions within 30 days.

**Conclusion:**

Point-of-care testing and its interpretation differed between the prescriber groups. Focus on interpreting point-of-care test results could be a way forward in antibiotic stewardship.

## KEY POINTS

- High prescribers used antibiotics at lower CRP levels and were more likely to identify a potential bacterial diagnosis.
- Many physicians reduced their antibiotic prescribing during the study period. Nine out of ten low prescribers remained low prescribers.
- Seeing a low-prescribing physician did not lead to more return visits or antibiotic changes.

## Introduction

Since antimicrobial resistance is a severe threat to global public health, the World Health Organization adopted in 2015 a global action plan [1]. The reduction of over-prescribing of antibiotics is an important factor in limiting antibiotic resistance [2]. However, there is still a considerable variation between physicians, regions, and countries in the use of antibiotics for infections in primary care, indicating that the optimal level is not yet reached [3–7].

High antibiotic prescribing has been associated with older physicians, higher patient volume, and longer time in practice [8–11]. High antibiotic prescribing frequency has also been associated with rural primary health care centres [11] and areas with low socioeconomic status [12]. However, these factors have limited explanatory power. Therefore, other factors such as diagnostic uncertainty, perceived severity of the illness, patients’ expectations, physicians’ perceptions of patients’ expectations, and communication skills have been suggested [13]. Nonetheless, the reasons for different antibiotic prescribing habits amongst physicians are unclear.

In the last three decades, there has been a decrease in antibiotic prescribing in Swedish primary care, especially for respiratory tract infections (RTI) and for children [7]. Antibiotic stewardship, pneumococcal conjugate vaccination, and public awareness are possible explanations [14,15]. It is unknown whether there is an even reduction in antibiotic prescribing in primary care among all physicians or if only some physicians have reduced their prescribing.

Most studies on antibiotic prescribing have been either cross-sectional or qualitative [13]. No study has, over time, compared physicians who reduce their prescribing of antibiotics to those who stay high prescribers. Understanding why some physicians continue to be high prescribers could facilitate future interventions.

The aim was to compare primary care physicians who, over time, reduced their antibiotic prescribing for respiratory tract infections with those who remained either high or low prescribers regarding potentially influencing factors. Primarily, factors influencing antibiotic prescribing rates were investigated such as physicians’ characteristics, the use of point-of-care tests and the choice of diagnoses. Secondly, the consequences of the different antibiotic prescribing rates were investigated, including return visit rate and renewed antibiotic prescribing.

## Materials and methods

The data in the present study have been extracted from a larger dataset, the Kronoberg Infection Database of Primary Care (KIDPC) [14]. In summary, the KIDPC dataset features all infection visits and all antibiotic treatments in primary care at 33 primary health care centres (PHCCs) and three out-of-hours offices in Kronoberg Region, Sweden, 2006–2014. During each visit, the physicians must register at least one diagnostic code according to the 10th revision of the International Statistical Classification of Diseases and Related Health Problems (ICD-10) or its modified Swedish PHC edition (KSH97-P). RTIs consist of the following diagnosis groups: acute bronchitis, acute media otitis, exacerbation of COPD, influenza, pharyngotonsillitis, pneumonia, sinusitis, upper RTI, and other RTIs (Supplemental Table 1). Antibiotic prescriptions were identified according to Anatomical Therapeutic Chemical Classification (ATC) code group J01, which includes all oral and parenteral antibiotics but not antibiotics in ointments or eye drops. Antibiotic prescriptions were included if linked to RTI diagnoses, i.e. if prescribed on the same day and at the same PHCC. The data in the KIDPC were extracted from the electronic medical records in Kronoberg County (Cambio Cosmic software, Cambio Healthcare Systems AB, Linköping, Sweden) on one occasion in 2015 using Business Objects (SAP AG, Walldorf, Germany).

In the present study, data on RTI visits were extracted from the KIDPC database, including information about the patient (age, sex), the physician (age, sex, training level), the PHCC, the investigations (C-reactive protein (CRP) test and rapid antigen detection test (RADT) for Group A Streptococci (GAS)), and the antibiotic treatment.

The data were divided into three 3-year periods. All 166 physicians who had diagnosed at least one RTI during each of the three periods were identified. On the other hand, 847 physicians had not been active during all three periods and were excluded at this stage. These were locums, interns not continuing in family medicine, and physicians who moved or retired.

The antibiotic prescribing rate was defined as the number of antibiotic prescriptions at RTI visits divided by the number of RTI visits. The antibiotic prescribing rates for RTIs per 3-year period were calculated for each physician and were adjusted for the patients’ sex and age group.

The physicians were divided into prescriber groups in three steps. Firstly, they were classified into three equal levels based on their antibiotic prescribing rate during the first period, 2006–2008. Low-level prescribers were defined as having an antibiotic prescribing rate below 40%, medium-level prescribers as having an antibiotic prescribing rate between 40 and 48%, and high-level prescribers as having an antibiotic prescribing rate over 48%. Secondly, during the third period, 2012–2014, the physicians were again divided into three levels using the same cut-offs.

**Figure 1.**
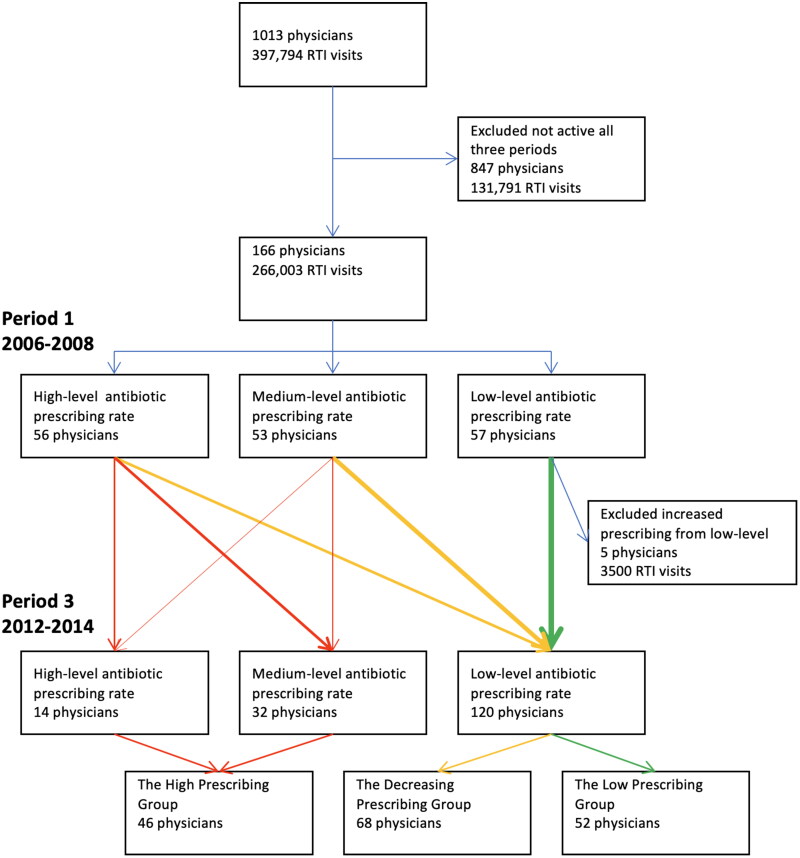
Flow chart showing the inclusion process and division into prescriber groups based on change of antibiotic prescription rate for respiratory tract infections (RTI) between the first period 2006–2009 and the third period 2012–2014.

Finally, in the third step, three prescriber groups were identified: The High Prescribing Group (consisting of high- or medium-level prescribers during both the first and the third period), the Decreasing Prescribing Group (consisting of high- or medium-level prescribers during the first period who transitioned to low-level prescribers during the third period), the Low Prescribing Group (consisting of low-level prescribers during both the first and the third period). Five physicians who were low-level prescribers during the first period and were medium- or high-level prescribers during the third period were excluded from further analyses as they did not fit in with the predefined prescriber groups (Figure 1).

The remaining 161 physicians were included and had 263,000 RTI visits, corresponding to 66% of all RTI visits in the region. In all, they had prescribed 108,000 antibiotic prescriptions at RTI visits, corresponding to 66% of all RTI antibiotic prescriptions in the region (Table 1).

**Table 1.** Background information on primary care physicians, respiratory tract infections, and antibiotic prescriptions for included and excluded physicians during the study period 2006–2014.

		Physicians	
		Included	Excluded
		*n* = 161	*n* = 852
Physicians		*n* (%)	*n* (%)
Sex	Female	63 (39)	169 (20)
	Male	88 (55)	194 (23)
	Unknown	10 (6)	489 (57)
Birth year	≤1940s	34 (21)	43 (5)
	1950s	45 (28)	43 (5)
	1960s	45 (28)	65 (8)
	1970s	24 (15)	88 (10)
	>1980s	3 (2)	124 (15)
	Unknown	10 (6)	489 (57)
Training level			
Specialist in family medicine during	100% of visits	70 (43)	273 (32)
	50 to <100% of visits	41 (25)	38 (4)
	1 to <50% of visits	30 (19)	32 (4)
Junior physician or other specialist		20 (12)	509 (60)
Continuity – number of primary health care centres at which each physician has worked	1	28 (17)	564 (66)
	2	43 (27)	186 (22)
	3–4	50 (31)	80 (9)
	5+	40 (25)	22 (3)
**Respiratory tract infections**		***n* (% of RTI visits)**	***n* (% of RTI visits)**
Total visits		262,503 (100)	135,291 (100)
Out-of-hours visits		40,431 (15)	11,757 (9)
Index visits		220,979 (83)	114,469 (87)
Antibiotic prescriptions		107,767 (41)	56,509 (42)
Antibiotic prescriptions at index visits		91,859 (35)	48,463 (37)
Point-of-care testing for respiratory tract infections			
C-reactive protein test		104,361 (40)	60,665 (45)
Rapid antigen detection test for Group A *Streptococci*		50,253 (19)	33,584 (25)
**Number of respiratory tract infections visits per physician**			
		**RTI visits, *n***	**RTI visits, *n***
	10th percentile	259	6
	median	1630	52
	90th percentile	2925	406

RTI: respiratory tract infection.

The following characteristics of the physicians were used in the analyses: sex, birthyear, training level (specialist in family medicine during 0%, 1–49%, 50–99% or 100% of the infection visits), continuity (number of PHCC at which each physician has worked), out-of-hours rate (out-of-hours visits per total number of visits per physician), and activity level (total number of RTI visits).

An RTI visit was defined as an index visit if there was no RTI visit in the previous 30 days. A return visit was defined as an RTI visit within 1–30 days of an earlier RTI visit. Antibiotics at return visits were defined if antibiotics were prescribed at a return visit within 30 days of an index visit. These measures were linked to the physician of the index visit. The use and result of point-of-care tests (CRP and RADT) and diagnoses at index visits were also measured. These measures are reported in two ways: (1) numbers per index visits per prescriber group (group level), and (2) numbers per physician per prescriber group (physician level). In the latter case, the data were divided into quartiles.

Continuous variables with non-normal distribution were presented as medians (interquartile range, IQR), and the Median test was used to compare medians across groups. Continuous variables with normal distribution were presented as means with standard deviation (SD), proportions and rates per physician. The characteristics of the physicians, investigations, diagnoses, treatment, and follow-up were analysed at the group level using Pearson’s *χ*^2^ test to compare groups and Cramer’s V to measure the effect size. At the physician level, the data were divided into quartiles and the prescriber groups were compared using Pearson’s *χ*^2^ test. If the comparisons of the three prescriber groups were statistically significant, pairwise comparisons were conducted using Bonferroni correction (multiplying *p* values with three) to account for multiple analyses. To compare the High Prescribing Group with the Decreasing Prescribing Group, multiple logistic regression with a full model was performed using background factors (physicians’ sex and birth year) and selected variables that were significant in a univariate logistic regression as independent variables. All statistical analyses were performed using IBM SPSS Statistics (version 27). *p* Values <0.05 were considered significant.

## Results

### General development

There was a general reduction in antibiotic prescribing for RTI per physician. The mean adjusted antibiotic prescribing rate for RTI per physician decreased from 45% (SD 16%) during the first period to 35% (SD 13%) during the third period. When comparing the first and third periods 84% (139/166) of the prescribers decreased their antibiotic prescribing rate. The Decreasing Prescribing Group consisted of 62% (68/109) of the medium- and high-level prescribers during the first period that became low-level prescribers in the third period. The High Prescribing Group consisted of 38% (41/109) of the medium- and high-level prescribers during the first period who remained medium- or high-level prescribers in the third period. Finally, the Low Prescribing Group consisted of 91% (52/57) of the low-level prescribers during the first period who remained low-level prescribers in the third period, and 9% (5/57) of the low-level prescribers during the first period became medium- or high-level prescribers in the third period and were excluded from further analyses. See Figure 1.

### Characteristics of physician

The only significant difference when comparing the three prescriber groups was the number of RTI visits, where a lower frequency was more common in the Low Prescribing Group. No significant differences were found in physicians’ sex, birth year, training level, continuity, and out-of-hours work (Table 2).

**Table 2.** Comparison of characteristics of the primary care physicians in the prescriber groups (the Low Prescribing Group, the Decreasing Prescribing Group, and the High Prescribing Group).

		Prescriber groups			
		Low Prescribing Group	Decreasing Prescribing Group	High Prescribing Group	*p*-Value
Primary care physicians’ characteristics		*n* (%)	*n* (%)	*n* (%)	
Physicians’ sex	Female	16 (36)	31 (46)	16 (41)	0.53
	Male	29 (64)	36 (54)	23 (59)	
Physicians’ birth year	≤1940s	13 (29)	12 (18)	9 (23)	0.28
	1950s	9 (20)	26 (39)	10 (26)	
	1960s	13 (29)	17 (25)	15 (38)	
	≥1970s	10 (22)	12 (18)	5 (13)	
Training Level					
Specialist in Family Medicine	100% of visits	20 (38)	36 (53)	14 (34)	0.24
	50 to <100% of visits	14 (27)	15 (22)	12 (29)	
	1 to <50% of visits	8 (15)	11 (16)	11 (27)	
Junior physician or other specialist		10 (19)	6 (9)	4 (10)	
Physicians’ continuity – number of primary health care centres at which each physician has worked	1	12 (23)	10 (15)	6 (15)	0.3
	2	16 (31)	21 (31)	6 (15)	
	3-4	14 (27)	23 (34)	13 (32)	
	≥5	10 (19)	14 (21)	16 (39)	
Physicians’ out-of-hours rate – Out-of-hours visits per total number of visits per physician	0%	13 (25)	5 (7)	7 (17)	0.084
	0.1 to <10%	13 (25)	16 (24)	13 (32)	
	10 to <20%	19 (37)	32 (47)	11 (27)	
	≥20%	7 (13)	15 (22)	10 (24)	
Physicians’ activity level - number of respiratory tract infection visits in total per physician	≤900	23 (44)	9 (13)	8 (20)	0.006^*^
	901–1600	8 (15)	19 (28)	12 (29)	
	1601–2300	14 (27)	20 (29)	12 (29)	
	>2300	7 (13)	20 (29)	9 (22)	

The variables are based on data for all nine years and assigned to the physician of the index visit.

*The comparison between the Low Prescribing Group and the Decreasing Prescribing Group was significant at *p* < 0.001. No other pairwise comparison was significant.

### Point-of-care testing

All the analyses of point-of-care testing were limited to index visits.

At the group level, CRP tests were analysed in 36% of the index visits in the Low Prescribing Group, 39% in the Decreasing Prescribing Group, and 44% in the High Prescribing Group (*p* < 0.001, Cramer’s V 0.06). Between the first and the third period the CRP test use increased from 37 to 43% of the index visits, and the increase was observed in all three prescriber groups. The median CRP values for all CRP tests were the same (10 mg/L) for the three prescriber groups (*p* = 0.073), but when antibiotics were prescribed the median CRP values differed: 45 mg/L (IQR 15–82) in the Low Prescribing Group, 33 mg/L (IQR 10–67) in the Decreasing Prescribing Group, and 23 mg/L (IQR 6–53) in the High Prescribing Group (*p* < 0.001). The result was similar although not significant when limiting to index visits with a diagnosis of pneumonia.

Rapid antigen detection test (RADT) for Group A Streptococci (GAS) was used in 20% of the index visits at the group level. The RADT use (tests per index visit) was 16% in the Low Prescribing Group, 19% in the Decreasing Prescribing Group, and 26% in the High Prescribing Group. 95% of cases with positive RADT received antibiotics (*p* < 0.001, Cramer’s V 0.09). Between the first and the third period, the use of RADT increased from 20 to 21% during the index visits, where the increase was observed in the High Prescribing Group but not in the Low Prescribing Group. Patients with a negative RADT also received antibiotics in some cases: 15% of cases with negative RADT in the Low Prescribing Group, 22% in the Decreasing Prescribing Group, and 35% in the High Prescribing Group (*p* < 0.001, Cramer’s V 0.175). The result was also significant when limiting to index visits, resulting in a diagnosis of pharyngotonsillitis.

### Diagnoses

At the group level, the diagnosis code for upper RTI was selected for index visits in 46% of the Low Prescribing Group, 38% in the Decreasing Prescribing Group, and 29% in the High Prescribing Group (*p* < 0.001, Cramer’s V 0.13). Between the first and the third period, the diagnosis of upper RTI increased from 37 to 38% of the index visits. There was an increase in the Decreasing Prescribing Group (from 37 to 38%) and the High Prescribing Group (from 27 to 31%) but a reduction in the Low Prescribing Group (from 48 to 44%). An opposite pattern was seen for pharyngotonsillitis, acute media otitis and sinusitis where the diagnoses were more frequent in the High Prescribing Group (Supplemental Table 2).

### Follow-up

Return visits within 30 days occurred in 14% of the Low Prescribing Group, 14% in the Decreasing Prescribing Group, and 15% in the High Prescribing Group (*p* < 0.001, Cramer’s V 0.009). If antibiotics were prescribed at the index visit, return visits occurred in 16% of index visits in all prescriber groups (*p* = 0.64).

Antibiotics were prescribed a second time within 30 days in 6.0% of the index visits in the Low Prescribing Group, 6.1% in the Decreasing Prescribing Group, and 6.6% in the High Prescribing Group (*p* < 0.001, Cramer’s V 0.01). If antibiotics were prescribed at the index visit, there was no difference in the proportion of second antibiotic prescriptions between the prescriber groups (*p* = 0.40).

### Comparison at the physician level

The use and result of different tests, the selection of diagnoses, and the rate of return visits and antibiotics within 30 days are shown per physician per prescriber group in Table 3. Compared with the group level the pattern was similar. Prescribers belonging to the High Prescribing Group used antibiotics at lower median CRP values and treated more patients with negative RADT for GAS with antibiotics. They were also more likely to select a diagnosis with potential bacterial aetiology. The Decreasing Prescribing Group had a lower return visit rate within 30 days compared to the High Prescribing Group. The antibiotic prescribing rate at return visits within 30 days was similar between the groups.

**Table 3.** Comparison of the prescriber groups (the Low Prescribing Group, the Decreasing Prescribing Group, and the High Prescribing Group) by physicians’ use of point-of-care tests, their choice of diagnosis, and how often the patients returned and received antibiotics.

		Prescriber groups			*p*-Value			
		Low Prescribing Group *n* (%)	Decreasing Prescribing Group *n* (%)	High Prescribing Group *n* (%)	All	Low vs. Decreasing Prescribing Group^a^	Decreasing vs. High Prescribing Group^a^	Low vs. High Prescribing Group^a^
CRP test use at index visits (% of index visits)	<26%	17 (33)	17 (25)	7 (17)	0.463			
	26 to <40.2%	12 (23)	19 (28)	9 (22)				
	40.2 to <52.7%	9 (17)	17 (25)	14 (34)				
	≥52.7%	14 (27)	15 (22)	11 (27)				
Median CRP test result at index visits per physician (mg/L)	<9.5	14 (27)	22 (32)	14 (34)	0.881			
	9.5–11.5	17 (33)	25 (37)	16 (39)				
	12	6 (12)	7 (10)	4 (10)				
	≥13	15 (29)	14 (21)	7 (17)				
Median CRP test result at index visits per physician where antibiotics were prescribed (mg/L)	<24	6 (12)	14 (21)	22 (54)	<0.001	0.022	0.001	<0.001
	24–36	6 (12)	20 (29)	14 (34)				
	37–46	17 (33)	21 (31)	3 (7)				
	≥46.5	23 (44)	13 (19)	2 (5)				
Median CRP test result at index visits with pneumonia where antibiotics were prescribed per physician (mg/L)	<59	11 (22)	12 (18)	17 (42)	0.059			
	59–75	10 (20)	19 (28)	10 (25)				
	75.5–96.5	16 (32)	19 (28)	4 (10)				
	≥97	13 (26)	17 (25)	9 (22)				
RADT use for GAS at index visits	<13%	19 (37)	18 (26)	4 (10)	0.001	1.000	0.021	0.001
	13 to <19%	11 (21)	19 (28)	10 (24)				
	19 to <25.9%	15 (29)	18 (26)	7 (17)				
	≥25.9%	7 (13)	13 (15)	20 (49)				
Negative RADT result for GAS at index visits where antibiotics were prescribed (% of tested)	<23%	20 (38)	19 (28)	2 (5)	<0.001	1.000	<0.001	<0.001
	23 to <34%	15 (29)	19 (28)	5 (12)				
	34 to <48%	9 (17)	20 (29)	12 (29)				
	≥48%	8 (15)	10 (15)	22 (54)				
Negative RADT result for GAS at index visits with pharyngotonsillitis where antibiotics were prescribed (% of tested)	<11%	22 (42)	13 (19)	5 (11)	0.001	0.153	0.099	0.003
	11 to <23%	13 (25)	22 (32)	5 (11)				
	23 to <33%	9 (17)	18 (26)	17 (39)				
	≥33	8 (15)	15 (22)	17 (39)				
The likelihood of selecting a diagnosis with potential bacterial aetiology^b^ (% of index visits)	<33%	27 (52)	12 (18)	2 (5)	<0.001	<0.001	<0.001	<0.001
	33.0 to <37.3%	17 (33)	22 (32)	1 (2)				
	37.3 to <43.5%	4 (8)	27 (40)	9 (22)				
	≥43.5%	4 (8)	7 (10)	29 (71)				
Return visits within 30 days (% of index visits)	<13.0%	15 (29)	15 (22)	11 (27)	0.029	0.696	0.015	1.000
	13.0 to <14.3%	13 (25)	19 (28)	7 (17)				
	14.3 to <15.3%	11 (21)	24 (35)	6 (15)				
	≥15.3%	13 (25)	10 (15)	17 (41)				
Antibiotics at return visits within 30 days (% of index visits)	<5.4%	15 (29)	16 (24)	10 (24)	0.419			
	5.4 to <6.1%	10 (19)	22 (32)	8 (20)				
	6.1 to <6.8%	16 (31)	15 (22)	9 (22)				
	≥6.8%	11 (21)	15 (22)	14 (34)				

The variables are based on data for all nine years and assigned to the physician of the index visit. The variables are divided into quartiles.

CRP: C-reactive protein; GAS: Group A *Streptococci*; RADT: rapid antigen detection test.

^a^*p*-Value adjusted for pairwise comparison with Bonferroni correction by multiplying with three.

^b^Diagnoses with potential bacterial aetiology: acute media otitis, pharyngotonsillitis, pneumonia, and sinusitis.

### Comparison of the Decreasing Prescribing Group and the High Prescribing Group

To study factors of importance for belonging to the Decreasing Prescribing Group compared to the High Prescribing Group we included in a multiple regression model the physicians’ age, sex, and continuity, as well as the variables that emerged as significant in the groupwise comparison. Odds ratios for belonging to the High Prescribing Group compared to the Decreasing Prescribing Group (index group) were analysed. Two variables remained significant in the adjusted model: Physicians in the High Prescribing Group were more likely to select a diagnosis with potential bacterial aetiology and to prescribe antibiotics to patients with negative RADT for GAS (Table 4). A sensitivity analysis where physicians with less than 50 RTI visits were omitted showed similar results.

**Table 4.** Association between physician characteristics and belonging to the high prescribing group as compared to the decreasing prescribing group.

	Crude model		Adjusted model	
	OR	95% CI	OR	95% CI
Male	1.24	0.56–2.75	0.53	0.11–2.48
Birth year	0.99	0.96–1.03	0.96	0.89–1.03
Number of PHCC	1.28	1.06–1.54	1.20	0.86–1.66
Median CRP level if antibiotics (mg/L)	0.92	0.88–0.96	0.99	0.93–1.06
Negative RADT treated with antibiotics (%)	1.08	1.04–1.11	1.08	1.02–1.14
Potential bacterial diagnoses (% of index visits)	1.31	1.18–1.46	1.32	1.15–1.52

The variables are based on data for all nine years and assigned to the physician of the index visit. Crude and adjusted odds ratio (ORs) with 95% confidence intervals (95% CI). Adjusted ORs were calculated using multiple logistic regressions with a full model.

Number of PHCC – the number of primary health care centres the physician has worked at. Median CRP level if antibiotics – the median C-reactive protein level for the physicians’ patients at index visits who were prescribed antibiotics. Negative RADT treated with antibiotics – the proportion of patients where rapid antigen detection test for Group A *Streptococci* was performed with negative results and still prescribed antibiotics. Potential bacterial diagnoses – acute media otitis, pharyngotonsillitis, pneumonia, and sinusitis.

## Discussion

This register-based study showed differences between the prescriber groups regarding the use and interpretation of point-of-care tests and the likelihood of registering a diagnosis with potential bacterial aetiology. Compared to the Low Prescribing Group, the High Prescribing Group ordered more CRP testing, prescribed antibiotics at lower CRP levels, ordered more RADT for GAS, and prescribed antibiotics more often when negative RADT. Also, the High Prescribing Group was more prone to register a diagnosis with potential bacterial aetiology than the Low Prescribing Group. Regarding these parameters, the Decreasing Prescribing Group was between the High and the Low Prescribing Group.

The lower antibiotic prescribing rate at index visits in the Low Prescribing Group did not result in more return visits or antibiotic prescriptions within 30 days. There were no differences in physicians’ characteristics between the prescriber groups besides that having few RTI visits was more common in the Low Prescribing Group.

### Strengths

The physicians were followed up for nine years at individual and group levels. During the study period, the same electronic medical record system was used in all the PHCCs in the region, including out-of-hours offices. The dataset is, therefore, comprehensive for primary care in the region. The study was performed before the development of telehealth services, which means that the risk of missing visits made in other regions via telehealth services is low. It would be difficult to replicate the study today. 78% of all antibiotic prescriptions were linkable to an infection diagnosis, which is a high level compared to a study from England [14,16].

### Limitations

Other models could have been chosen to divide the physicians into groups. However, the aim was to study physicians who reduced their antibiotic prescribing rate. Thirteen physicians had less than ten RTI visits in either the first or the third period which makes the antibiotic prescribing rate inexact (Four physicians had less than five RTI visits in either period). Information regarding age and sex is missing for a few physicians, and this could affect the absence of differences. Possible confounders such as the patients’ comorbidity, and smoking habits are missing in this dataset. Some prescriptions were missing due to being prescribed in a dose-dispensing system without connection to the electronic medical records, which mainly affects prescriptions to some elderly patients (75 years and older).

The dataset was collected prior to the introduction of telehealth medicine and before the onset of the COVID-19 pandemic, which may limit its relevance to current healthcare practices. However, concerning factors that influence the physicians’ antibiotic prescribing, we believe that this study is still relevant, since the differences between prescriber groups, the choices of point-of-care use and its interpretation as well as the choice of diagnosis are not likely to be affected by telehealth services or post-pandemic healthcare.

Furthermore, the study included only 161 physicians from one region in Sweden with less than 200,000 inhabitants, thus lessening the generalizability of the results. However, these physicians who remained in the region and did not retire took care of two-thirds of all RTI visits. Also, a reduction in antibiotic prescribing has been seen in the whole country during the study years. Therefore, it would be reasonable to generalise to the rest of Sweden and to other low-prescribing countries.

### A general decrease in antibiotic prescribing

The reduction in antibiotic prescribing in the study was similar throughout Sweden [7] and in other countries such as Norway [17], Finland [18], England [19] and Denmark [20] during the same period, but not in Australia [21]. Possible explanations include introducing the pneumococcal vaccination programme for children in 2009, financial incentives for reaching targets at regional and PHCC level of reduced level of antibiotic prescriptions, public awareness of the disadvantages of antibiotics, and antibiotic stewardship. The programme Strama for national antibiotic stewardship has been running since 1995 with a wide range of actions: committed work at the local and national levels, monitoring antibiotic use, surveillance of resistance, raising awareness and behavioural change [15]. Consequently, the Decreasing Prescribing Group in this study was large.

### Characteristics of prescribers

Physicians with few RTI visits were more likely to belong to the Low Prescribing Group. The reason is unclear. They may have a patient population with a higher prevalence of chronic diseases and a lower incidence of acute infections. Alternatively, it might be attributed to random chance.

Physicians’ age did not affect the antibiotic prescribing rate in this study. However, an earlier study from Sweden has shown that older physicians are more prone to antibiotic prescribing [22], and a similar pattern has been reported in Canada, England, Germany, and the Netherlands [11,23–25]. Since only a few physicians increased their antibiotic prescribing over time, they seem more likely to maintain their prescribing pattern with increasing age. Perhaps the higher prescribing rate seen among older physicians in other studies reflects a higher general prescribing rate when the physicians were younger.

Locum physicians have sometimes been identified as high prescribers [26]. In this study, the locums belong to the exclusion group, which had the same antibiotic prescribing rate as the included physicians. Some studies have reported more high prescribers among physicians trained abroad [23,24]. Unfortunately, this study lacks information about the education country.

### Point-of-care tests

Although significantly different at the group level, the use of CRP test was not significantly different at the physician level amongst the prescriber groups. The use of RADT was more common in the High Prescribing Group than in the other prescriber groups both at group level and physician level. Also, the High Prescribing Group used antibiotics at lower CRP levels and more often when RADT was negative. It is unclear whether point-of-care tests decrease or increase antibiotic prescribing. In this study, the use of CRP testing was increasing while the antibiotic prescribing was decreasing. A similar pattern was seen in a Danish study of primary care [27]. A Cochrane review shows that CRP testing for acute respiratory tract infections reduces antibiotic prescribing [28]. Other studies show that RADT testing for GAS increases antibiotic prescribing [6,29,30].

It can be argued that the lower median CRP levels and the higher incidence of negative RADT observed in the High Prescribing Group when prescribing antibiotics may represent circular evidence. Assuming the patient populations are similar, this will follow if more antibiotics are prescribed. However, focusing on interpreting point-of-care results could be a way forward in antibiotic stewardship.

### Choice of diagnoses

In a Swedish context, the diagnosis ‘Upper RTI’ is considered to be of viral origin. Physicians in the Low Prescribing Group were more likely to diagnose upper RTI, while physicians in the High Prescribing Group were more likely to register a diagnosis with potential bacterial aetiology. The same pattern is seen in several other studies where the proportion of potential bacterial diagnoses corresponds to the antibiotic prescribing rate [11,31,32]. The assumption is that an infection is assigned a potential bacterial diagnosis to justify the use of antibiotics.

### Follow-up

Earlier studies have reported a rate of 27–38% of return visits within a month. Some have shown a higher rate of return visits if antibiotics were prescribed at the index visit, and others have shown the opposite [33–35]. The rate of return visits was generally lower (14%) in the current study. The low rate of return visits is probably due to generally fewer consultations per inhabitant. There is a small statistical difference in return visits amongst the prescriber groups, but the effect size is small, and the difference is clinically irrelevant. When comparing at the physician level, the only difference was a lower return visit rate in the Decreasing Prescribing Group compared to the High Prescribing Group.

The proportion of a second antibiotic prescription within 30 days was statistically significant between the prescriber groups, but again the effect size was small, and the difference lacks clinical significance. When comparing at the physician level no significance was seen.

### Other explanations

Generally, the physician is considered solely responsible for prescribing antibiotics, but other factors such as the impact of the PHCCs and patients’ expectations are relevant [11,13,36]. These factors have not been explored in this study.

Qualitative studies have identified several potential factors. A study of sore throat identified different strategies for physicians to cope with uncertainty: adherence to guidelines; clinical picture and CRP; expanded control; and unstructured examination [37]. In a study of lower RTI, physicians mentioned that fear of consequences was a reason for antibiotics [38]. In a recent study on acute sinusitis, physicians mentioned sympathy with the patient, contextual factors such as Fridays with limited possibility to follow-up, and the patient’s appearance and level of pain [39].

Physicians’ choice to prescribe antibiotics may be motivated by special circumstances (no possibility of follow-up; previous severe infections; close relation to an immunocompromised patient; or concurrent severe diseases). Perhaps physicians in the High Prescribing Group were more likely to identify special circumstances that motivated them to prescribe antibiotics.

### Implications

The study highlights potential factors to address regarding high antibiotic prescribers, such as the tendency to overuse point-of-care tests and the quality of the interpretation of tests. Furthermore, the result emphasises the importance of correct diagnosis to maintain high quality in antibiotic prescribing. The results could be useful in quality improvement in primary care, focusing on information, continuous medical education on indications, usefulness and interpretation of the point-of-care tests.

## Conclusion

The use and interpretation of point-of-care testing were different amongst the prescriber groups. The High Prescribing Group did more CRP testing, prescribed antibiotics at lower CRP levels, performed more RADT for GAS, prescribed antibiotics more often although negative RADT, and were more prone to register a diagnosis with potential bacterial aetiology than the Low Prescribing Group. The Decreasing Prescribing Group was in between the High Prescribing Group and the Low Prescribing Group regarding these variables. There was no clinically relevant difference in the proportions of return visits and new prescriptions of antibiotics within 30 days in the three prescriber groups. According to our results, focusing on the use and interpretation of point-of-care tests is a possible way to improve antibiotic stewardship.

## Supplementary Material

Supplemental Material

## Data Availability

The datasets used and analysed in the current study are available with the corresponding author upon reasonable request.

## References

[1] WHO. Global action plan on antimicrobial resistance. Geneva: WHO; 2015.

[2] Bell BG, Schellevis F, Stobberingh E, et al. A systematic review and meta-analysis of the effects of antibiotic consumption on antibiotic resistance. BMC Infect Dis. 2014;14(1):13. doi: 10.1186/1471-2334-14-13. 24405683 PMC3897982

[3] Cars O, Mölstad S, Melander A. Variation in antibiotic use in the European Union. Lancet. 2001;357(9271):1851–1853. doi: 10.1016/S0140-6736(00)04972-2. 11410197

[4] Bjerrum L, Boada A, Cots JM, et al. Respiratory tract infections in general practice: considerable differences in prescribing habits between general practitioners in Denmark and Spain. Eur J Clin Pharmacol. 2004;60(1):23–28. doi: 10.1007/s00228-003-0706-z. 14689127

[5] Tyrstrup M, van der Velden A, Engstrom S, et al. Antibiotic prescribing in relation to diagnoses and consultation rates in Belgium, The Netherlands and Sweden: use of European quality indicators. Scand J Prim Health Care. 2017;35(1):10–18. doi: 10.1080/02813432.2017.1288680. 28277045 PMC5361413

[6] van der Velden AW, van de Pol AC, Bongard E, et al. Point-of-care testing, antibiotic prescribing, and prescribing confidence for respiratory tract infections in primary care: a prospective audit in 18 European countries. BJGP Open. 2022;6(2):BJGPO.2021.0212. doi: 10.3399/BJGPO.2021.0212. 34920989 PMC9447323

[7] Swedres-Svarm. Sales of antibiotics and occurrence of antibiotic resistance in Sweden; 2021. Solna/Uppsala 2022. https://www.folkhalsomyndigheten.se/publikationer-och-material/publikationsarkiv/s/swedres-svarm-2021/

[8] Gjelstad S, Dalen I, Lindbaek M. GPs’ antibiotic prescription patterns for respiratory tract infections–still room for improvement. Scand J Prim Health Care. 2009;27(4):208–215. doi: 10.3109/02813430903438718. 19929185 PMC3413912

[9] Aspinall SL, Good CB, Metlay JP, et al. Antibiotic prescribing for presumed nonbacterial acute respiratory tract infections. Am J Emerg Med. 2009;27(5):544–551. doi: 10.1016/j.ajem.2008.04.015. 19497459

[10] Cadieux G, Tamblyn R, Dauphinee D, et al. Predictors of inappropriate antibiotic prescribing among primary care physicians. CMAJ. 2007;177(8):877–883. doi: 10.1503/cmaj.070151. 17923655 PMC1995156

[11] Hueber S, Kuehlein T, Gerlach R, et al. “What they see is what you get": prescribing antibiotics for respiratory tract infections in primary care: do high prescribers diagnose differently? An analysis of German routine data. PLOS One. 2017;12(12):e0188521. doi: 10.1371/journal.pone.0188521. 29220399 PMC5722345

[12] Koller D, Hoffmann F, Maier W, et al. Variation in antibiotic prescriptions: is area deprivation an explanation? Analysis of 1.2 million children in Germany. Infection. 2013;41(1):121–127. Feb doi: 10.1007/s15010-012-0302-1. 22826031

[13] O’Connor R, O’Doherty J, O’Regan A, et al. Antibiotic use for acute respiratory tract infections (ARTI) in primary care; what factors affect prescribing and why is it important? A narrative review. Ir J Med Sci. 2018;187(4):969–986. 29532292 10.1007/s11845-018-1774-5 PMC6209023

[14] Cronberg O, Tyrstrup M, Ekblom K, et al. Diagnosis-linked antibiotic prescribing in Swedish primary care – a comparison between in-hours and out-of-hours. BMC Infect Dis. 2020;20(1):616. doi: 10.1186/s12879-020-05334-7. 32819280 PMC7441551

[15] Mölstad S, Löfmark S, Carlin K, et al. Lessons learnt during 20 years of the Swedish strategic programme against antibiotic resistance. Bull World Health Organ. 2017;95(11):764–773. doi: 10.2471/BLT.16.184374. 29147057 PMC5677604

[16] Dolk FCK, Pouwels KB, Smith DRM, et al. Antibiotics in primary care in England: which antibiotics are prescribed and for which conditions? J Antimicrob Chemother. 2018;73(suppl_2):ii2–ii10. Feb 1 doi: 10.1093/jac/dkx504. 29490062 PMC5890730

[17] Skow M, Fossum GH, Høye S, et al. Antibiotic treatment of respiratory tract infections in adults in Norwegian general practice. JAC Antimicrob Resist. 2023;5(1):dlac135. 36632357 10.1093/jacamr/dlac135 PMC9825809

[18] Parviainen S, Saastamoinen L, Lauhio A, et al. Outpatient antibacterial use and costs in children and adolescents: a nationwide register-based study in Finland, 2008–16. J Antimicrob Chemother. 2019;74(8):2426–2433. doi: 10.1093/jac/dkz208. 31102531

[19] Bou-Antoun S, Costelloe C, Honeyford K, et al. Age-related decline in antibiotic prescribing for uncomplicated respiratory tract infections in primary care in England following the introduction of a national financial incentive (the quality premium) for health commissioners to reduce use of antibiotics in the community: an interrupted time series analysis. J Antimicrob Chemother. 2018;73(10):2883–2892. doi: 10.1093/jac/dky237. 29955785

[20] Kristensen PK, Johnsen SP, Thomsen RW. Decreasing trends, and geographical variation in outpatient antibiotic use: a population-based study in Central Denmark. BMC Infect Dis. 2019;19(1):337. doi: 10.1186/s12879-019-3964-9. 31014280 PMC6480614

[21] Andersson K, van Driel M, Hedin K, et al. Antibiotic use in Australian and Swedish primary care: a cross-country comparison. Scand J Prim Health Care. 2022;40(1):95–103. doi: 10.1080/02813432.2022.2036494. 35166180 PMC9090355

[22] Tell D, Engström S, Mölstad S. Adherence to guidelines on antibiotic treatment for respiratory tract infections in various categories of physicians: a retrospective cross-sectional study of data from electronic patient records. BMJ Open. 2015;5(7):e008096. doi: 10.1136/bmjopen-2015-008096. PMC4513445 26179648

[23] Silverman M, Povitz M, Sontrop JM, et al. Antibiotic prescribing for nonbacterial acute upper respiratory infections in elderly persons. Ann Intern Med. 2017;167(10):758–759. doi: 10.7326/L17-0438. 29159387

[24] Wang KY, Seed P, Schofield P, et al. Which practices are high antibiotic prescribers? A cross-sectional analysis. Br J Gen Pract. 2009;59(567):e315-20–e320. doi: 10.3399/bjgp09X472593. 19843411 PMC2751935

[25] Akkerman AE, Kuyvenhoven MM, van der Wouden JC, et al. Prescribing antibiotics for respiratory tract infections by GPs: management and prescriber characteristics. Br J Gen Pract. 2005;55(511):114–118. 15720932 PMC1463185

[26] Borek AJ, Pouwels KB, van Hecke O, et al. Role of locum GPs in antibiotic prescribing and stewardship: a mixed-methods study. Br J Gen Pract. 2022;72(715):e118–e127. doi: 10.3399/BJGP.2021.0354. 34990397 PMC8763197

[27] Sydenham RV, Justesen US, Hansen MP, et al. Prescribing antibiotics: the use of diagnostic tests in general practice. A register-based study. Scand J Prim Health Care. 2021;39(4):466–475. doi: 10.1080/02813432.2021.2004721. 34845954 PMC8725972

[28] Smedemark SA, Aabenhus R, Llor C, et al. Biomarkers as point-of-care tests to guide prescription of antibiotics in patients with acute respiratory infections in primary care. Cochrane Database Syst Rev. 2014;10(10):Cd010130. 10.1002/14651858.CD010130.pub3 PMC9575154 36250577

[29] Aabenhus R, Siersma V, Sandholdt H, et al. Identifying practice-related factors for high-volume prescribers of antibiotics in Danish general practice. J Antimicrob Chemother. 2017;72(8):2385–2391. doi: 10.1093/jac/dkx115. 28430992

[30] Strandberg EL, Brorsson A, André M, et al. Interacting factors associated with low antibiotic prescribing for respiratory tract infections in primary health care – a mixed methods study in Sweden. BMC Fam Pract. 2016;17(17):78. doi: 10.1186/s12875-016-0494-z. 27430895 PMC4950701

[31] André M, Vernby A, Odenholt I, et al. Diagnosis-prescribing surveys in 2000, 2002 and 2005 in Swedish general practice: consultations, diagnosis, diagnostics and treatment choices. Scand J Infect Dis. 2008;40(8):648–654. doi: 10.1080/00365540801932439. 18979603

[32] Hutchinson JM, Jelinski S, Hefferton D, et al. Role of diagnostic labeling in antibiotic prescription. Can Fam Physician. 2001;47:1217–1224. 11421050 PMC2018517

[33] Cals JW, Hood K, Aaftink N, et al. Predictors of patient-initiated reconsultation for lower respiratory tract infections in general practice. Br J Gen Pract. 2009;59(567):761–764. doi: 10.3399/bjgp09X472656. 19843422 PMC2751918

[34] Holmes WF, Macfarlane JT, Macfarlane RM, et al. The influence of antibiotics and other factors on reconsultation for acute lower respiratory tract illness in primary care. Br J Gen Pract. 1997;47(425):815–818. 9463983 PMC1410080

[35] Little P, Gould C, Williamson I, et al. Reattendance and complications in a randomised trial of prescribing strategies for sore throat: the medicalising effect of prescribing antibiotics. BMJ. 1997;315(7104):350–352. doi: 10.1136/bmj.315.7104.350. 9270458 PMC2127265

[36] Petursson P. GPs’ reasons for “non-pharmacological” prescribing of antibiotics. A phenomenological study. Scand J Prim Health Care. 2005;23(2):120–125. doi: 10.1080/02813430510018491. 16036552

[37] Andre M, Gröndal H, Strandberg EL, et al. Uncertainty in clinical practice – an interview study with Swedish GPs on patients with sore throat. BMC Fam Pract. 2016;17(17):56. doi: 10.1186/s12875-016-0452-9. 27188438 PMC4870808

[38] Bisgaard L, Andersen CA, Jensen MSA, et al. Danish GPs’ experiences when managing patients presenting to general practice with symptoms of acute lower respiratory tract infections: a qualitative study. Antibiotics. 2021;10(6):1–12. doi: 10.3390/antibiotics10060661. PMC8228607 34205866

[39] Thaulow J, Eide TB, Høye S, et al. Decisions regarding antibiotic prescribing for acute sinusitis in Norwegian general practice. A qualitative focus group study. Scand J Prim Health Care. 2023;41(4):469–477. doi: 10.1080/02813432.2023.2274328. 37902260 PMC11001307

