# Supplementary material for: Factors influencing antibiotic prescribing for respiratory tract infections in primary care – a comparison of physicians with different antibiotic prescribing rates
Source: Scand J Prim Health Care. 2024 Apr 16;42(3):424–34. doi: 10.1080/02813432.2024.2332757 (PMC11332303; doi:10.1080/02813432.2024.2332757)
Supplement: Supplemental Material [file IPRI_A_2332757_SM2060.docx]

**Supplement Table 1**. Respiratory tract infection (RTI) diagnoses in the Kronoberg Infection Database in Primary care 2006-2014. The total number of patients and RTI visits. The total number of diagnoses is 407,770 registered at 397.794 RTI visits, thus at some visits more than one diagnosis were registered,

| ICD-10 | Diagnosis code description | Subgroup | Number of patients | Number of visits |
| --- | --- | --- | --- | --- |
| A37- | Whooping cough | Other respiratory tract infection | 14 | 14 |
| A38- | Scarlet fever | Other respiratory tract infection | 1,063 | 1,120 |
| A389 | Scarlet fever, uncomplicated | Other respiratory tract infection | 1 | 1 |
| A691 | Other Vincent’s infections | Other respiratory tract infection | 48 | 48 |
| A70- | Chlamydia psittaci infections | Other respiratory tract infection | 4 | 4 |
| B27- | Infectious mononucleosis | Other respiratory tract infection | 1,467 | 1,809 |
| B270 | Gammaherpesviral mononucleosis | Other respiratory tract infection | 1 | 1 |
| B279 | Infectious mononucleosis, unspecified | Other respiratory tract infection | 2 | 2 |
| B34-P | Viral infection of unspecified site | Upper respiratory tract infection | 27,170 | 35,230 |
| B349 | Viral infection, unspecified | Upper respiratory tract infection | 15 | 15 |
| H65- | Nonsuppurative otitis media | Other respiratory tract infection | 8,136 | 9,649 |
| H653 | Chronic mucoid otitis media | Other respiratory tract infection | 3 | 3 |
| H660 | Acute suppurative otitis media | Acute otitis media | 22,859 | 41,310 |
| H661 | Chronic tubotympanic suppurative otitis media | Acute otitis media | 1 | 1 |
| H663P | Suppurative and unspecified otitis media | Acute otitis media | 196 | 230 |
| H669 | Otitis media, unspecified | Acute otitis media | 3 | 3 |
| H669P | Suppurative and unspecified otitis media | Acute otitis media | 5,919 | 7,736 |
| H70- | Mastoiditis and related conditions | Other respiratory tract infection | 57 | 60 |
| H72- | Perforation of tympanic membrane | Other respiratory tract infection | 743 | 857 |
| H730 | Acute myringitis | Other respiratory tract infection | 35 | 36 |
| J01- | Acute sinusitis | Sinusitis | 19,411 | 31,531 |
| J010 | Acute maxillary sinusitis | Sinusitis | 2 | 2 |
| J012 | Acute ethmoidal sinusitis | Sinusitis | 1 | 1 |
| J019 | Acute sinusitis, unspecified | Sinusitis | 5 | 5 |
| J02- | Acute pharyngitis | Pharyngotonsillitis | 7,403 | 8,159 |
| J029 | Acute pharyngitis, unspecified | Pharyngotonsillitis | 2 | 2 |
| J03- | Acute tonsillitis | Pharyngotonsillitis | 31,550 | 49,490 |
| J030 | Streptococcal tonsillitis | Pharyngotonsillitis | 1 | 1 |
| J039 | Acute tonsillitis, unspecified | Pharyngotonsillitis | 7 | 7 |
| J04- | Acute laryngitis and tracheitis | Other respiratory tract infection | 1,879 | 2,075 |
| J040 | Acute laryngitis | Other respiratory tract infection | 2 | 2 |
| J050 | Acute obstructive laryngitis [croup] | Other respiratory tract infection | 1,386 | 1,665 |
| J051 | Acute epiglottitis | Other respiratory tract infection | 63 | 64 |
| J06-P | Acute upper respiratory infections of multiple and unspecified sites | Upper respiratory tract infection | 68,800 | 114,396 |
| J060 | Acute laryngopharyngitis | Upper respiratory tract infection | 1 | 1 |
| J069 | Acute upper respiratory infection, unspecified | Upper respiratory tract infection | 29 | 29 |
| J099 | Influenza due to certain identified influenza viruses | Influenza | 67 | 76 |
| J101 | Influenza due to other identified influenza virus with other respiratory manifestations | Influenza | 1 | 1 |
| J11-P | Influenza due to unidentified influenza virus | Influenza | 3,466 | 3,709 |
| J157 | Pneumonia due to Mycoplasma pneumoniae | Pneumonia | 2 | 2 |
| J159 | Unspecified bacterial pneumonia | Pneumonia | 5 | 6 |
| J18-P | Pneumonia, unspecified organism | Pneumonia | 18,048 | 26,134 |
| J180 | Bronchopneumonia, unspecified organism | Pneumonia | 2 | 2 |
| J189 | Pneumonia, unspecified organism | Pneumonia | 13 | 13 |
| J209 | Acute bronchitis, unspecified | Acute bronchitis | 6 | 6 |
| J219 | Acute bronchiolitis, unspecified | Acute bronchitis | 2 | 2 |
| J22-P | Unspecified acute lower respiratory infection | Acute bronchitis | 30,476 | 42,652 |
| J229 | Unspecified acute lower respiratory infection | Acute bronchitis | 2 | 2 |
| J310 | Chronic rhinitis | Other respiratory tract infection | 1,051 | 1,174 |
| J312P | Chronic rhinitis, nasopharyngitis and pharyngitis | Other respiratory tract infection | 130 | 135 |
| J32- | Chronic sinusitis | Other respiratory tract infection | 467 | 548 |
| J320 | Chronic maxillary sinusitis | Other respiratory tract infection | 1 | 1 |
| J340 | Abscess, furuncle and carbuncle of nose | Other respiratory tract infection | 126 | 142 |
| J36- | Peritonsillar abscess | Other respiratory tract infection | 793 | 896 |
| J369 | Peritonsillar abscess | Other respiratory tract infection | 3 | 3 |
| J37- | Chronic laryngitis and laryngotracheitis | Other respiratory tract infection | 79 | 81 |
| J409 | Bronchitis, not specified as acute or chronic | Other respiratory tract infection | 2 | 2 |
| J410 | Simple chronic bronchitis | Other respiratory tract infection | 1 | 3 |
| J42-P | Unspecified chronic bronchitis | Other respiratory tract infection | 1,518 | 2,139 |
| J429 | Unspecified chronic bronchitis | Other respiratory tract infection | 2 | 2 |
| J43- | Emphysema | Other respiratory tract infection | 191 | 324 |
| J439 | Emphysema, unspecified | Other respiratory tract infection | 3 | 3 |
| J44- | Other chronic obstructive pulmonary disease | Chronic obstructive pulmonary disease | 5,922 | 23,910 |
| J440 | Chronic obstructive pulmonary disease with acute lower respiratory infection | Chronic obstructive pulmonary disease | 7 | 7 |
| J441 | Chronic obstructive pulmonary disease with (acute) exacerbation | Chronic obstructive pulmonary disease | 12 | 12 |
| J449 | Chronic obstructive pulmonary disease, unspecified | Other respiratory tract infection | 46 | 54 |
| J47- | Bronchiectasis | Other respiratory tract infection | 94 | 167 |
| J479 | Bronchiectasis, uncomplicated | Other respiratory tract infection | 3 | 3 |

**Supplement Table 2**. Number of diagnoses (percentage within each column) for respiratory tract infections at the index visits by prescriber groups (the Low Prescribing Group, the Decreasing Prescribing Group and the High Prescribing Group), total index visits (including index visits to excluded physicians), and total visits (all index and return visits).

|  |  |  |  |  |  | |  | |  |  |  |
| --- | --- | --- | --- | --- | --- | --- | --- | --- | --- | --- | --- |
|  |  | Index visits per prescriber group | | | | | | Total index visits | | | Total visits |
|  |  | Low Prescribing Group | Decreasing Prescribing Group | High Prescribing Group | | Total | |  | | |  |
| Acute bronchitis |  | 4821 (8.6) | 11,905 (11.2) | 6266 (10.7) | | 22,992 (10.4) | | 33,623 (10.0) | | | 40,601 (10.2) |
| Acute media otitis^b^ |  | 4937 (8.8) | 12,242 (11.5) | 8974 (15.4) | | 26,153 (11.8) | | 39,418 (11.5) | | | 48,107 (12.1) |
| Exacerbation of COPD |  | 3297 (5.9) | 7491 (7.0) | 3041 (5.2) | | 13,829 (6.3) | | 18,687 (5.6) | | | 23,058 (5.8) |
| Influenza^a^ |  | 670 (1.2) | 1185 (1.1) | 449 (0.8) | | 2304 (1.0) | | 3276 (1.0) | | | 3691 (0.9) |
| Pharyngotonsillitis^b^ |  | 6782 (12.1) | 13,912 (13.1) | 10,260 (17.6) | | 30,954 (14.0) | | 49,095 (14.6) | | | 56,675 (14.2) |
| Pneumonia^b^ |  | 2968 (5.3) | 5688 (5.3) | 3748 (6.4) | | 12,404 (5.6) | | 18,815 (5.6) | | | 25,749 (6.5) |
| Sinusitis^b^ |  | 3353 (6.0) | 7854 (7.4) | 5831 (10.0) | | 17,038 (7.7) | | 26,060 (7.8) | | | 30.962 (7.8) |
| URTI^a^ |  | 26,128 (46.4) | 40,292 (37.9) | 17,138 (29.4) | | 83,558 (37.8) | | 129,461 (38.6) | | | 146,706 (36.9) |
| Other RTI |  | 3317 (5.9) | 5788 (5.4) | 2642 (4.5) | | 11,747 (5.3) | | 18,013 (5.4) | | | 22,245 (5.6) |
| Total |  | 56,273 (100) | 106,357 (100) | 58,349 (100) | | 220,979 (100) | | 335,448 (100) | | | 397,794 (100) |

^a^ viral diagnosis. ^b^ potential bacterial diagnosis.

COPD Chronic obstructive pulmonary disease; RTI respiratory tract infection; URTI uppe
